# Epidemiological survey to determine the prevalence of cholecystolithiasis in Uyghur, Kazakh, and Han Ethnic Groups in the Xinjiang Uyghur Autonomous Region of China: cross-sectional studies

**DOI:** 10.1186/s12876-021-01677-w

**Published:** 2021-03-18

**Authors:** Fujun Lv, Guangjun Wang, Dandan Ding, Caifu Shen, Jiangwei Liu, Feng Ji, Yan Kang

**Affiliations:** 1 Department of Surgery, Kaifeng Central Hospital, Kaifeng, 475000 Henan Province China; 2 Department of General Surgery, General Hospital of Xinjiang Military Command, Urumqi, 830000 Xinjiang Uygur Autonomous Region China; 3 Department of Obstetrics, Kaifeng Obstetrics and Gynecology Hospital, Kaifeng, 475000 Henan Province China; 4 The 69240 Army Hospital of PLA, Xinjiang, Ürümqi, 830000 China

**Keywords:** Cholecystolithiasis, Prevalence, Risk factors, Epidemiology

## Abstract

**Background:**

This study was performed to understand the prevalence of and possible risk factors for cholecystolithiasis in Uyghur, Kazakh, Han, and other ethnic groups in the Xinjiang Uyghur autonomous region of China.

**Methods:**

Subjects were enrolled using typical case sampling and multistage stratified random sampling. We collected epidemiological data regarding cholecystolithiasis using a standard questionnaire of risk factors for gallbladder disease in Xinjiang. The subjects completed the questionnaire and underwent an abdominal ultrasound examination of the liver and gallbladder.

**Results:**

This study included 5454 Xinjiang residents aged ≥ 18 years. The prevalence of cholecystolithiasis was 15% (11.3% in men and 17.1% in women), and the sex difference was statistically significant (male-to-female odds ratio [OR] 1.867; *p* < 0.001). The cholecystolithiasis prevalence was also significantly different among the Han, Uyghur, Kazakh, and other ethnic groups (13.1%, 20.8%, 11.5%, and 16.8%, respectively; *p* < 0.001). The prevalence of cholecystolithiasis in northern Xinjiang was 13.5% and that in southern Xinjiang was 17.5%; this difference was also statistically significant (OR 1.599; *p* < 0.001). Across all ethnic groups, the cholecystolithiasis prevalence significantly increased with age (all *p* < 0.01) and body mass index (BMI) (all *p* < 0.01). A multivariate logistic regression analysis indicated that cholecystolithiasis prevalence was associated with sex, age, BMI, smoking, diabetes, fatty liver disease, and geographical differences between northern and southern Xinjiang.

**Conclusions:**

The prevalence of cholecystolithiasis was significantly higher in the Uyghur ethnic group than in the Han, Kazakh, and other ethnic groups; in women than in men; in southern Xinjiang than in northern Xinjiang; in patients with fatty liver disease; and increased with age and BMI. Our findings could provide a theoretical basis for the formulation of control measures for cholecystolithiasis.

**Supplementary Information:**

The online version contains supplementary material available at 10.1186/s12876-021-01677-w.

## Background

Cholecystolithiasis is a common clinical disease worldwide. It is a major public health problem in most Western countries, and it is more prevalent in those countries than in Asian countries. According to Acalovschi [1], the prevalence rates of cholecystolithiasis in Europe, Asia, South America, North America, and Africa are 5.9–21.9%, 3.2–10.7%, 13.3–17.9%, 14.3–28.5%, and 5.2%, respectively. The prevalence of cholecystolithiasis is increasing with the increase in the standard of living in China, but study results have varied. Survey data for China in 1989 showed that the detection rate of cholecystolithiasis was 6.6% overall; however, it varied between 3 and 15% in different regions [2]. An epidemiological survey of cholecystolithiasis in China from 2007 to 2010 showed that the prevalence rates in the populations of Taiwan [3] and Shanghai [4] were 5.3% and 10.7%, respectively. However, a randomized epidemiological survey of the Wensu County of Xinjiang [5] found a prevalence rate of 13.2%, which is much higher than the average prevalence of 10% in China.

Xinjiang comprises one-sixth of the total land area of China and has a high incidence of cholecystolithiasis. It is a vast area with multiple ethnic groups. However, no large-scale epidemiological data have comprehensively reflected the characteristics and incidence of cholecystolithiasis among all ethnic groups and all regions of Xinjiang. Therefore, prevention and treatment of cholecystolithiasis for the ethnic minorities in Xinjiang are impeded. This study collected epidemiological data regarding Uyghur, Kazakh, and Han populations in various regions of Xinjiang to analyze differences related to cholecystolithiasis, especially those regarding the prevalence of the disease. We also analyzed risk factors that may affect the prevalence of cholecystolithiasis in multiple ethnic groups that have not been reported in China or abroad.

This study aimed to explore the prevalence of, possible risk factors for, and epidemiological characteristics of cholecystolithiasis in the Uyghur, Kazakh, Han, and other ethnic groups in the Xinjiang Uyghur autonomous region of China.

### Methods

#### Epidemiological surveys and ethics statement

This study enrolled adults aged ≥ 18 years residing in Xinjiang for ≥ 1 year. Temporary migrant workers and those whose family members were registered in the region but have resided for less than 1 year were excluded. Those who did not undergo abdominal ultrasonography and did not complete the questionnaire were also excluded.

We used typical case sampling and multistage stratified random sampling methods. The first stage of stratification was based on the county as the sampling area, the second stage was based on the township as the sampling area, and the third stage was based on the village as the sampling area. Finally, the households comprised the individual samples. The township health centers (hospitals) served as platforms for surveying urban areas, and the community service centers (health centers) were platforms for surveying rural communities. Nine prefectures were selected according to the ethnic distribution in Xinjiang. Typical sampling was first performed to select the ethnic minority communities in various states, including six cities, 11 counties, and one Alatai Beitun division. Thereafter, multistage stratified random sampling was performed to select 23 natural villages and 22 communities. Because Uyghur, Kazakh, and Han individuals rarely marry outside their ethnic groups, mixed races are rare. If there are mixed races, then that is noted on the Chinese identity card. Uygur is the main ethnic group in southern Xinjiang, Kazak is the main ethnic group in northern Xinjiang, and Han is more widely distributed.

We designed our own questionnaire (see Additional file 1) for this study. The questionnaire was also translated into the Uyghur language. From June 2011 to September 2011, we conducted the survey based on a sampling ratio of approximately 1:1 of the rural and urban areas. The plan was to randomly select 38 households in each village and community (approximately 150 respondents) and 20 households in certain small villages (approximately 100 respondents). The village cadres or the staff of the community service centers guided our team to each household; hence, we could administer the survey questionnaires and schedule appointments for the hepatobiliary ultrasound examination.

#### Survey items

The questionnaire included the following main items: (1) general information such as sex, age, weight, height, and place of residence; (2) smoking habits (at least 1 cigarette daily for 6 months) and alcohol consumption (at least 100 g three times per week for 6 months); (3) history of diabetes and fatty liver disease; and (4) ultrasound examination.

According to the World Health Organization recommendations for weight classifications for Asians [6], BMI was classified as follows: normal range, 18.5–22.9 kg/cm^2^; normal, > 23 kg/cm^2^; overweight, 23–24.9 kg/cm^2^; obese, 25–29.9 kg/cm^2^; and extremely obese, ≥ 30 kg/cm^2^.

#### Diagnostic criteria

The diagnostic criteria for cholecystolithiasis were based on the following ultrasound results: appearance of a morphologically stable and strong echogenic mass in the gallbladder lumen with posterior acoustic shadowing and movement of the echogenic mass toward the direction of gravity with changes in body position; disappearance of the sound-permeable fluid lumen of the normal gallbladder; and presence of an arc-shaped or semi-moon-shaped intermediate or strong echogenic strand on the front wall of the outline of the gallbladder with wide posterior acoustic shadowing. Ultrasonography showed no gallbladder or abdominal cholecystectomy scar, and postoperative pathology confirmed the gallstone.

Ultrasound is known as a safe, accurate, and convenient tool for diagnosing cholecystolithiasis, with high sensitivity (97%) and specificity (93.6%) [7]. It can accurately diagnose cholecystolithiasis in approximately 96% of patients [8].

#### Quality control

Ultrasound examinations of the gallbladder were performed by two associate chief physicians of the ultrasound department of the General Hospital of Xinjiang Military Command using a digital portable ultrasound machine [9, 14] (Mindray DP-6600; probe frequency, 3.5 MHz; Shenzhen, China).

All staff members who participated in the study were healthcare professionals from the General Hospital of Xinjiang Military Command. They had a good understanding of the research area, were well-trained in administering the questionnaires, and had conducted some preliminary surveys during the study period. The preliminary survey was used to select residents in the military residential area to complete the questionnaire. During the survey process, the questionnaire was constantly improved and modified, including the questioning methods, procedures of the investigators and verification of portable ultrasound. During the investigation, the investigators and respondents completed the questionnaires together face to face, and it was ensured that they were complete. Incomplete questionnaires were followed-up to obtain missing information. In addition, 5% of the sample was selected for review, and the accuracy rate reached 99.5%.

Ten medical health officers from a military medical team in Turfan served as clerks who performed data entry. Before data entry, the clerks received strict group training. Data were entered twice, and differences between the two entries were compared and corrected to ensure accuracy. The average time required for each investigator and respondent to complete the questionnaire was 9.37 min. The degree of cooperation when completing the questionnaire was analyzed. The rate of satisfactory cooperation was 98.6%, whereas the rate of unsatisfactory cooperation was 1.4%. Respondents who exhibited poor cooperation when completing the questionnaires were considered lost to follow-up.

#### Enrollment of patients with cholecystolithiasis, fatty liver, and type 2 diabetes

Selected participants were instructed to fast overnight (for at least 7–8 h) and were to undergo an upper abdominal ultrasound examination at their local health center or community health services station during a scheduled period. A unified questionnaire that contained personal information (weight, height, age, sex) and information regarding history of type 2 diabetes, alcohol consumption, smoking habits, district of residence, family history of gallbladder diseases, and dietary habits was collected from the participants. However, the diagnosis of type 2 diabetes mainly depended on hospital validation (county-based and by larger institutions). Fatty liver was detected by ultrasound examination of the participants' livers. Body mass index (BMI) was calculated as body weight in kilograms divided by the square of body height in meters (kg/m^2^).

#### Statistical analysis

EpiData 3.1 was used to enter data from qualified questionnaires twice and to check for errors. Then, data were imported to SPSS 25.0, where data analysis was performed. Possible risk factors were first analyzed using univariate logistic regression, and the statistically significant factors were analyzed using multifactor logistic regression. The chi-squared test was used to compare two or more sample rates, and α = 0.05 was used as the significance level of the tests.

### Results

#### Characteristics of participants

A total of 6024 respondents from Xinjiang participated in the survey; of these, 5454 fulfilled the inclusion criteria. The response rate was 90.54%. There were 1946 (35.7%) male and 3508 (64.3%) female participants; The average age was 45.2 ± 14.1 years; the youngest participant was aged 18 years and the eldest 88 years. Only 14 participants had both cholecystolithiasis and gallbladder polyps, but no statistical analyses were performed. To be clearer, here a baseline characteristics table (see Additional file 1).

#### Prevalence of cholecystolithiasis in Xinjiang

The prevalence of cholecystolithiasis in Xinjiang was 15%. Based on different ethnic groups, the prevalence were 13.1% for Hans, 20.8% for Uyghurs, 11.5% for Kazakhs, and 16.8% for other ethnic groups; these were all statistically significant (*p* < 0.001). The prevalence were 13.5% in northern Xinjiang and 17.5% in southern Xinjiang; these were also statistically significant (*p* < 0.001).

#### Prevalence of cholecystolithiasis in different ethnic groups and age groups according to sex

**Fig. 1:**
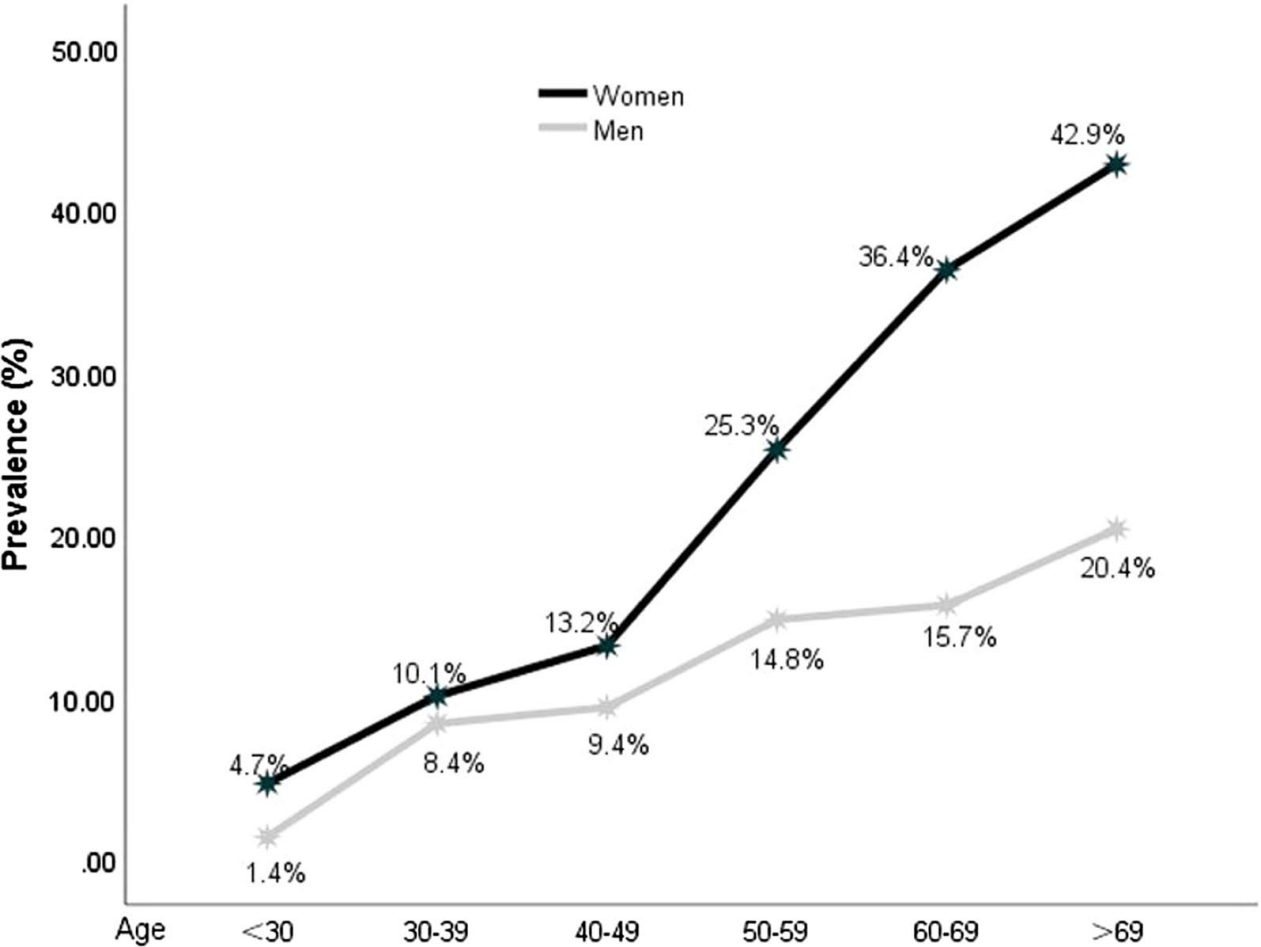
Prevalence of cholecystolithiasis for different age groups according to sex

The prevalence of cholecystolithiasis were 11.3% in men and 17.1% in women; these were statistically significant (*p* < 0.001). The prevalence of cholecystolithiasis for men and women of all ethnic groups significantly increased with age (both *p* < 0.01) (Fig. 1). Differences in the prevalence of cholecystolithiasis based on sex were statistically significant in the Han, Uyghur, and other ethnic groups (all *p* < 0.05), but not in the Kazakh ethnic group (Table 1).

**Table 1:** Prevalence of cholecystolithiasis for different ethnic groups according to sex

Sex	Hans	Uyghurs	Kazakhs	Other ethnic groups
	Prevalence (%)	Prevalence (%)	Prevalence (%)	Prevalence (%)
Men	79/896 (8.8)	70/483 (14.5)	54/416 (13.0)	16/151 (10.6)
Women	219/1378 (15.9)	222/923 (24.1)	94/871 (10.8)	66/336 (19.6)
Total	298/2274 (13.1)	292/1406 (20.8)	148/1287 (11.5)	82/487 (16.8)
*p* value	0.001	0.001	0.263	0.013

#### Prevalence of cholecystolithiasis for different ethnic groups according to age

The prevalence of cholecystolithiasis significantly increased with age in all ethnic groups (all *p* < 0.01). Furthermore, the prevalence of all age groups across all ethnic groups were statistically significant (all *p* < 0.05). In particular, the prevalence of cholecystolithiasis for each age group in Uyghurs were higher than those in the Han, Kazakh, and other ethnic groups (Table 2).

**Table 2:** Prevalence of cholecystolithiasis for different ethnic groups according to age

Age group (years)	Hans	Uyghurs	Kazakhs	Other ethnic groups	*p* value
	Prevalence (%)	Prevalence (%)	Prevalence (%)	Prevalence (%)	
< 30	5/201 (2.5)	10/180 (5.6)	4/231 (1.7)	6/66 (9.1)	0.015
30–39	28/444 (6.3)	59/363 (16.3)	33/473 (7.0)	17/148 (11.5)	0.001
40–49	69/734 (9.4)	47/373 (12.6)	36/276 (13.0)	27/133 (20.3)	0.003
50–59	67/371 (18.1)	66/221 (29.9)	33/165 (20.0)	14/83 (16.9)	0.004
60–69	80/338 (23.7)	74/187 (39.6)	21/881 (23.9)	11/42 (26.2)	0.001
> 69	49/186 (26.3)	36/82 (43.9)	21/54 (38.9)	7/15 (46.7)	0.016
Total	298/2274 (13.1)	292/1406 (20.8)	148/1287 (11.5)	82/487 (16.8)	0.001

#### Prevalence of cholecystolithiasis for different ethnic groups according to BMI

Differences in the cholecystolithiasis prevalence among all ethnic groups were statistically significant among all BMI groups (< 23, 23–24.5, and > 25 kg/cm^2^) (all *p* < 0.01). The prevalence increased with increasing BMI in the Han, Kazakh, and Uyghur ethnic groups (*p* < 0.001), but not in other ethnic groups. The prevalence of cholecystolithiasis for all BMI groups among the Uyghurs group were higher than those for all BMI groups among the Han, Kazakh, and other ethnic groups (Table 3).

**Table 3:** Prevalence of cholecystolithiasis for different ethnic groups according to BMI

BMI group (kg/cm^2^)	Hans	Uyghurs	Kazakhs	Other ethnic groups	*p* value	Total
	Prevalence (%)	Prevalence (%)	Prevalence (%)	Prevalence (%)		Prevalence (%)
< 23	96/965 (9.9)	92/652 (14.1)	38/573 (6.6)	31/203 (15.3)	0.001	257/2393 (10.7)
23–24.9	85/586 (14.5)	46/232 (19.8)	20/240 (8.3)	16/115 (13.9)	0.005	167/1173 (14.2)
≥ 25	117/723 (16.2)	154/522 (29.5)	90/474 (19.0)	35/169 (20.7)	0.001	396/1888 (21.0)
Total	298/2274 (13.1)	292/1406 (20.8)	148/1287 (11.5)	82/487 (16.8)	0.001	820/5454 (15.0)

#### Prevalence of cholecystolithiasis for patients with and without fatty liver disease

**Fig. 2:**
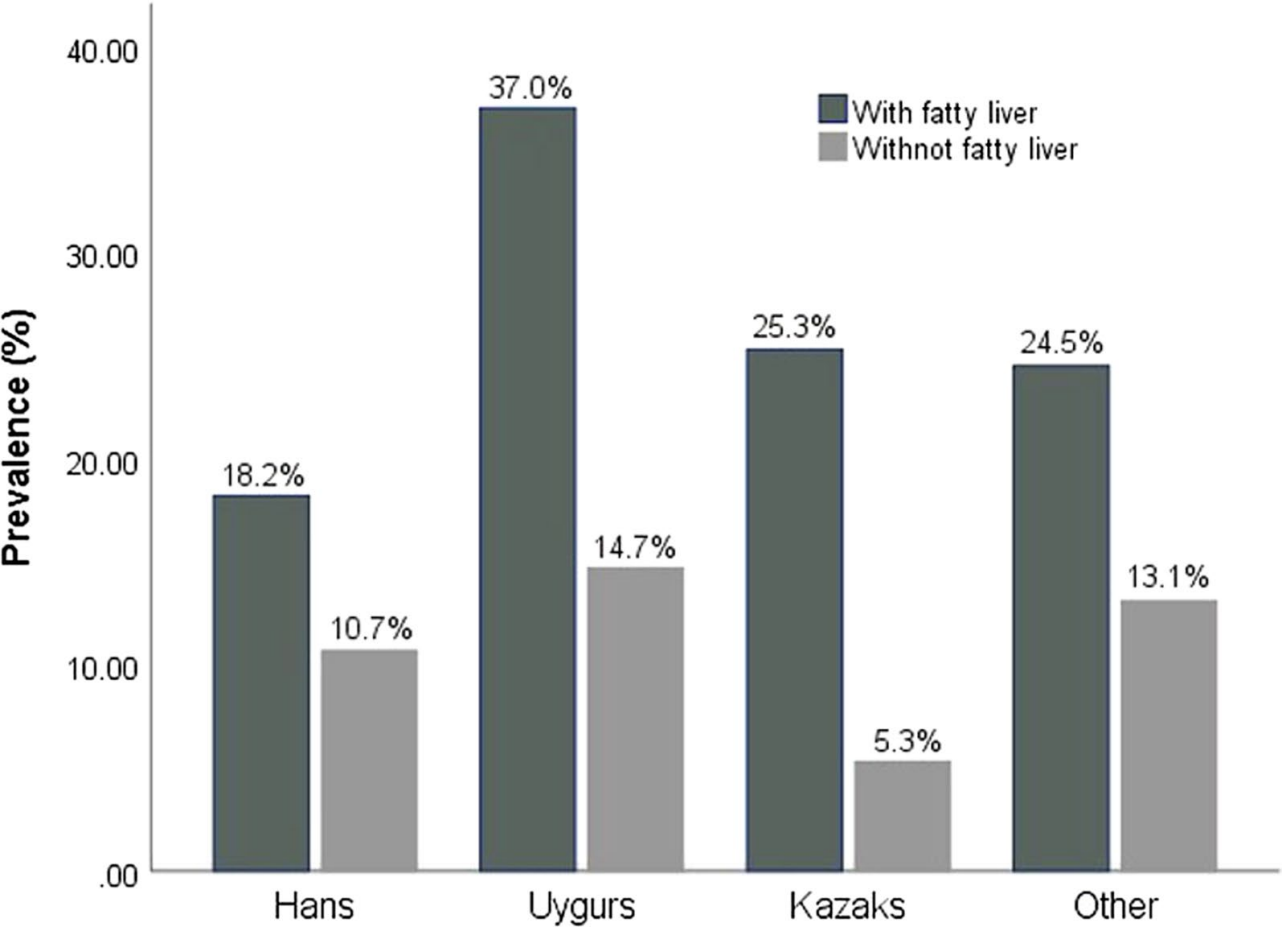
Prevalence of cholecystolithiasis for patients with and without fatty liver disease

Among all ethnic groups in this study, the prevalence of cholecystolithiasis was significantly higher in patients with fatty liver disease than in those without, and the differences were statistically significant (all *p* < 0.01). The Uyghur population exhibited the highest prevalence among all ethnic groups, followed by Kazakh (Fig. 2).

#### Multivariate logistic regression analysis of factors related to cholecystolithiasis

A multivariate logistic regression analysis was performed for factors related to cholecystolithiasis that were statistically significant in the univariate analysis. These factors were incorporated in the model after adjusting for other confounders (Table 4). Alcohol consumption is not statistically significant and is not entered in the multivariate logistic regression analysis of factors. The analysis revealed that sex, age, BMI, smoking habits, diabetes, fatty liver disease, and regional differences between northern and southern Xinjiang, but not alcohol consumption, were associated with the prevalence of cholecystolithiasis. Cholecystolithiasis prevalence was higher among women, older adults, high BMI, non-smoking habits, history of diabetes, history of fatty liver disease, and residents living in Southern Xinjiang.

**Table 4:** Results of the multivariate logistic regression analysis of factors related to cholecystolithiasis

Factors	Coefficient B	SE	Wald χ^2^	*df*	*p* value	OR	95% CI	
Sex	0.624	0.109	32.944	1	0.001	1.867	1.508	2.310
Age	0.046	0.003	235.688	1	0.001	1.047	1.041	1.053
BMI	0.041	0.013	10.363	1	0.001	1.042	1.016	1.068
Smoking habits	− 0.291	0.110	7.007	1	0.008	0.747	0.602	0.927
History of diabetes	0.567	0.152	13.979	1	0.001	1.763	1.310	2.372
History of fatty liver disease	0.764	0.094	66.585	1	0.001	2.148	1.788	2.581
Northern versus southern Xinjiang	0.469	0.082	32.612	1	0.001	1.599	1.361	1.879
Ethnicity								
Han								
Uyghur	0.650	0.100	41.919	1	0.001	1.916	1.574	2.333
Kazakh	− 0.149	0.107	1.930	1	0.165	0.862	0.698	1.063
Other	0.295	0.136	4.684	1	0.030	1.343	1.028	1.753
Constant	− 8.065	0.496	264.918	1	0.001	0.001		

Age and BMI were continuous variables, Classification variables of related factors (see Additional file 1). Odds ratio (OR) = 1, no effect on cholecystolithiasis; OR > 1, risk factor; OR < 1, protective factor

## Discussion

To our knowledge, no large-scale study on the prevalence of cholecystolithiasis among ethnic minorities in Xinjiang has been performed. The survey data were mainly collected from the Han, Uygur, and Kazak ethnic groups. The prevalence of cholecystolithiasis ranged from 5.9 to 21.9% in the West and from 3.1 to 10.7% in Asia [10]. We also found a significant difference in the prevalence of northern and southern Xinjiang in China. In other regions of China, the prevalence of cholecystolithiasis ranges from 4.2 to 12.1% [11–13], and a randomized epidemiological survey of Wensu County in Xinjiang [5] found that the prevalence was as high as 13.2%. The main finding of this study was that the prevalence of cholecystolithiasis was significantly higher in Uyghurs (20.8%) than in Hans (13.1%), Kazakhs (11.5%), and other ethnic groups (16.8%).

A meta-analysis [1] showed that the prevalence of gallbladder stones varied greatly among states. Differences in prevalence among countries or states also exist because of factors such as living environments, dietary habits, and culture. In our study, we found that the lifestyle habits of Uyghurs were significantly different from those of other ethnic groups in Xinjiang. Within the same ethnic groups investigated, we previously [14] found that the consumption of fresh fruits and vegetables was a protective factor against cholecystolithiasis. Misciagna [15] showed that physical activity and consumption of unsaturated fatty acids could reduce the risk of gallstone formation. Uyghurs prefer high-fat (saturated fatty acid), high-protein, and high-sugar foods such as beef, lamb, milk, tea, cheese, and candies. During the long winters (winters in Xinjiang can last up to 6 months), they rarely eat fresh fruits and vegetables and exercise less frequently. It was speculated that the climate of Xinjiang is characterized by large temperature differences between day and night, which is typical of the dry temperate continental climate. However, this factor requires further investigation. Additionally, these differences may be attributable to genetic factors among Uyghurs.

Our results showed that the prevalence of cholecystolithiasis was higher in women than in men in Han, Uyghur, and other ethnic groups [11, 16]. However, recent research has shown inconsistent findings. Zhu et al. [17] reported that the prevalence of cholecystolithiasis in only the Uyghur population is related to sex; this is not true for the Han population. In our study, this was unanimously determined for the Uyghur population but not for the Han population. This may be related to the scope of the data collection. The prevalence in Kazakhs is not related to sex, and the prevalence in Kazakh women is low. Furthermore, Kazakhs, including women, are mostly nomadic, have high activity levels, and usually eat more horsemeat, which is rich in unsaturated fatty acids [18]. The low prevalence of cholecystolithiasis related to their lifestyle and dietary habits [15] may be affected by both ethnicity and sex. However, the significant increase in the prevalence of cholecystolithiasis among Uyghur women may be related to their tendency to gain body weight after marriage.

Most studies have indicated that age is related to the prevalence of cholecystolithiasis [17, 19]. The prevalence of cholecystolithiasis was highest in 60–69 years old [10]. Our study showed that age is an important factor that affects the prevalence of cholecystolithiasis among ethnic minorities in Xinjiang. With increasing age, the prevalence increased, especially among women. Additionally, in our study, the prevalence of cholecystolithiasis in the population aged 30 to 39 years increased significantly. Among Uyghurs, the prevalence for those aged 30 to 39 years was 2.9- to 7.8-times higher than that for those aged < 30 years. For Hans, the prevalence was 2.5- to 10.5-times higher for those aged < 30 years. Moreover, for Kazakhs, the prevalence was 4.1- to 22.9-times higher for those aged < 30 years. It was found that the prevalence of cholecystolithiasis for the population aged 30 to 39 years increased by a similar factor among the Han and Uyghur ethnic groups. Although the overall prevalence of cholecystolithiasis in the Kazakh ethnic group was not high, it increased by the most obvious factor for those aged 30 to 39 years. Age differences between ethnic groups in relation to cholecystolithiasis have not been reported in the relevant literature. It has been speculated that the Kazakh ethnic group may be affected by external exposure risk factors. Southern Xinjiang is dominated by Uyghurs and has more solar radiation, higher temperatures, less rainfall, and experience more drought conditions than northern Xinjiang, which is dominated by Kazakhs. Most Kazaks are nomads who move with their herds. Their exposure to risk factors for cholecystolithiasis tends to begin to increase between ages 30 and 39 years. This gradual increase of exposure to cholecystolithiasis risk factors can also explain the increased prevalence of disease in the elderly.

Obesity is also related to the formation of cholecystolithiasis [17] and increases the risk of related complications [20]. The most common method used to assess obesity is BMI. In our study, the prevalence of cholecystolithiasis for ethnic minorities with BMI > 25 kg/cm^2^ increased significantly. The highest prevalence of the disease was found for Uyghurs with obesity; their prevalence rate was 1.82-times and 1.56-times higher than those of Hans and Kazakhs, respectively. The prevalence rates of cholecystolithiasis in the Han, Uyghur, and Kazakh obese populations were 1.6-, 2.1-, and 2.8-times that of the normal population. According to the multivariate logistic regression analysis, BMI remained an influencing factor for cholecystolithiasis in the three ethnic groups, but obesity was not an independent factor. A previous study indicated that the prevalence of obesity was higher in the Uyghur and Kazakh populations [21]. The role of obesity in gallstone formation might be related to the activity of rate-limiting enzymes, which increases cholesterol synthesis in the liver and promotes cholesterol oversaturation and secretion to the bile ducts, thereby inhibiting peristalsis of the bile ducts and leading to the formation of cholecystolithiasis [22].

Substantial evidence has proven that fatty liver disease is closely associated with the cholecystolithiasis. The prevalence of cholecystolithiasis significantly increased in patients with fatty liver disease, especially alcoholic fatty liver disease [23–25]. However, the relationship between alcoholic fatty liver disease and non-alcoholic fatty liver disease was not determined during our survey. Our study showed that the prevalence of cholecystolithiasis in the Han, Uyghur, and Kazakh populations with fatty liver disease were 18.2% (133/597), 37% (142/242), and 25.3% (101/29), respectively (*p* < 0.001). Therefore, the prevalence of cholecystolithiasis in people with fatty liver disease were higher than that of the normal population by 1.39-times (18.2% vs 13.1% for Hans), 1.78-times (37% vs 20.8% for Uyghurs), and 2.20-times (25.3% vs 11.5% for Kazakhs). Furthermore, diabetes may also contribute to an increased prevalence of cholecystolithiasis. The multivariate logistic regression analysis indicated that fatty liver disease (OR 2.148) and diabetes (OR 1.763) were associated with the formation of cholecystolithiasis. Diabetes has been shown to promote gallstone formation among the ethnic minorities of Xinjiang, and other reports have shown a positive correlation between diabetes mellitus and gallstone formation [26–28]. However, the mechanisms by which fatty liver disease and diabetes promote gallstone formation remain unclear.

As mentioned, the prevalence of cholecystolithiasis for Uyghurs was high. In addition to obesity and age, genetics could be related to the high prevalence in the Uyghur population. However, the overall prevalence was low among Kazakhs; this could also be due to external environmental and physical factors such as obesity, age, and living environments. In Korea [29], the prevalence of cholecystolithiasis among immigrants residing on Jeju Island is relatively high. The majority of Han people living in Xinjiang are immigrants. This type of emigration phenomenon suggests that the prevalence of cholecystolithiasis in the Han ethnic group is affected by their acquired living environment. Residing in Xinjiang may have changed their dietary habits, living environment, climate, and biological clock. The time difference between Xinjiang and the outer region has delayed the breakfast time of those who migrated to Xinjiang by approximately 2.5 h, leading to asynchronization in the peak time of hormone secretion. Other studies have suggested that smoking habits may not be associated with the risk of cholecystolithiasis in men and that alcohol is postulated to prevent gallstone formation [30]. However, our results suggest the contrary. A multivariate logistic regression analysis found that smoking habits, but not alcohol consumption, were associated with cholecystolithiasis. These inconsistent results may be related to the different populations investigated; therefore, further studies are warranted.

Our results indicate that the prevalence of cholecystolithiasis in Uyghurs was very high; it was approximately three times higher than that reported in the United States in 2011 [31]. The prevalence was significantly lower in Kazakhs, who also live in Xinjiang, and significantly higher in Hans who migrated to Xinjiang than in those in other parts of China. The high-fat diet of Kazakhs and Uyghurs may promote gallstone formation [32], but most Kazakhs live a nomadic life and their main form of transportation is the horse. Such a physical activity and reduced sitting time may be responsible for reducing the risk of cholecystolithiasis [33]. Although Kazakhs do not consume many fresh fruits and vegetables, they often consume nuts and onions. Nuts are rich in vitamin E, which can prevent cholecystolithiasis [34], and traditional Chinese medicine has suggested that onions have blood lipid-lowering effects. Our study showed that factors such as sex, age, BMI, smoking habits, diabetes, fatty liver disease, and regional differences between northern and southern Xinjiang were associated with the prevalence of cholecystolithiasis in the different ethnic groups. However, we could not ignore the genetic differences in these ethnic groups. Previous studies have also shown that gallstone formation is related to some major genetic factors [35]. Therefore, risk factors for cholecystolithiasis among the ethnic minorities in Xinjiang remain a topic worthy of further investigation.

This study had some limitations. First, ethnic minorities other than the Uyghurs and Kazakhs comprise relatively small populations; therefore, they could not be discussed extensively during the study. Therefore, our results cannot represent the prevalence of cholecystolithiasis for other ethnic minorities and cannot comprehensively represent the prevalence for all minorities in Xinjiang. Second, although color Doppler ultrasound has high sensitivity and accuracy for detecting fatty liver disease, it cannot distinguish between alcoholic fatty liver and non-alcoholic fatty liver. Third, the diagnosis of diabetes was only based on data from local medical centers instead of the results of multiple blood tests conducted by our own team. Many smokers smoked only small amounts, which may have affected the results of the analysis. Weight was measured by weighing instruments of different medical units, which may have varied; however, the difference is within 300 g. Finally, many factors affecting cholecystolithiasis were not included in this study; therefore, further studied are necessary.

## Conclusions

Our study showed that the prevalence of cholecystolithiasis was significantly higher in Uyghurs than in the Han, Kazakh, and other ethnic groups; in women than in men; and in people residing in southern Xinjiang than in northern Xinjiang. Additionally, the prevalence of cholecystolithiasis increased with age in the Han, Uygur, Kazakh, and other ethnic groups, and it was positively correlated with BMI in the Han, Kazakh, and Uyghur populations. Uyghurs had higher prevalence of cholecystolithiasis than among all ethnic groups in age, sex, BMI and fatty liver disease groups. Moreover, the multivariate logistic regression analysis revealed that sex, age, BMI, smoking habits, diabetes, fatty liver disease, and regional differences between northern and southern Xinjiang were associated with the prevalence of cholecystolithiasis. Our findings could provide a theoretical basis for the formulation of control measures for cholecystolithiasis and could serve as a guide for improving the awareness, prevention, and treatment of cholecystolithiasis in various ethnic groups in Xinjiang.

## Supplementary Information

**Additional file 1**. Supplementary documents on basic population characteristics, classified variable data and questionnaires.

## Data Availability

(1) The datasets used during the current study are available from the corresponding author on reasonable request. (2) [Figshare] repository: https://doi.org/10.6084/m9.figshare.13108100, link: https://figshare.com/.

## Abbreviations

BMI: Body mass index
CI: Confidence interval
*df*: Degree of freedom
OR: Odds ratio
SE: Standard error
