# Supplementary material for: Epidemiological survey to determine the prevalence of cholecystolithiasis in Uyghur, Kazakh, and Han Ethnic Groups in the Xinjiang Uyghur Autonomous Region of China: cross-sectional studies
Source: BMC Gastroenterol. 2021 Mar 18;21:125. doi: 10.1186/s12876-021-01677-w (PMC7977306; doi:10.1186/s12876-021-01677-w)
Supplement: Supplementary file 1 — Additional file 1. Supplementary documents on basic population characteristics, classified variable data and questionnaires. [file 12876_2021_1677_MOESM1_ESM.docx]

**Table 1 Basic characteristics of the populations**

|  | Hans | Uyghurs | Kazakhs | Other ethnic groups |
| --- | --- | --- | --- | --- |
| Age | 48.01±13.93 | 45.29±14.31 | 41.04±13.35 | 43.13±12.86 |
| Body mass index | 23.75±3.15 | 23.92±3.89 | 24.05±4.05 | 24.09±3.60 |
| Northern  Xinjiang | 1396 | 480 | 1270 | 210 |
| Southern Xinjiang | 878 | 926 | 17 | 277 |
| Married  Unmarried  Other Marital Status | 1990(87.5%)  137(6.0%)  147(6.5%) | 1195(85.0%)  95(6.8%)  116(8.3%) | 1090(84.7%)  141(11%)  56(4.4%) | 426(87.5%)  41(8.4%)  20(4.1%) |

**Table 2** **Classification variables of related factors**

| Related factors | Classification |
| --- | --- |
| Sex | 1=male, 2=female |
| Xinjiang location | 1=Northern Xinjiang, 2=southern Xinjiang |
| Alcohol consumption | 1=no, 2=yes |
| Smoking habits | 1=no, 2=yes |
| History of fatty liver | 1=no, 2=yes |
| History of diabetes | 1=no, 2=yes |
| Ethnicity | Dummy variable: Han (0,0,0); Uyghurs (1,0,0); Kazakhs (0,1,0); other ethnic groups (0,0,1) |

**Note: Age and BMI are continuous variables, and no values are required.**

**A Questionnaire on risk Factors of gallbladder disease in Xinjiang Uygur Autonomous Region**

Questionnaire number

Householder：

Name of respondent：

telephone number：

Family address：

After the investigation, verify the items in the form:

1 Questionnaire 1 incomplete 2 complete

2 Ultrasonography 1 incomplete 2 complete

3 cooperation 1 Very good 2 good 3 general 4 poor

4 Completion time: minutes

Investigator's name：

Survey area：

Survey date：

**Informed consent**

You are participating in the Xinjiang Uygur Autonomous Region Science and Technology Project, which is the "Study on Strategies for the Prevention and Control of Gallbladder Diseases in Xinjiang". The purpose of the questionnaire is to discover the reasons for the high incidence of gallbladder stones and polyps of various ethnic groups in Xinjiang, and to understand the prevalence of gallbladder diseases in various ethnic groups. Provide relevant theoretical support and scientific basis in the treatment and prevention of such diseases, and do a good job in the prevention and control of gallbladder diseases, reduce the incidence of gallbladder diseases and the occurrence of complications, and truly benefit all Xinjiang Ethnic people. The questionnaire survey needs to ask about your family and personal general situation, smoking, drinking, eating, sickness, and your ultrasound results.

All participants in this research project are voluntary, all inspections are free, all information about you will be strictly confidential, and will never be disclosed or made public without your own consent. Data information is only used for research and paper.

Before signing this consent form, I have read or heard others read the above content to me, have ample opportunity to consult and get a satisfactory answer. I participated in this investigation and physical examination voluntarily.

Thank you for participation!

Signature of survey subject：

Date of Signature:

Investigator’s signature：

Date of Signature:

**Attachment: Description of diet classification**

1． Fresh beans: soybean sprouts, mung bean sprouts, bean sprouts, kidney beans, lentils, peas, snow peas, cowpeas, fresh broad beans, edamame, etc.

2． Dried beans and products: mung beans, soybeans, red beans, soy milk, oily tofu, tofu, other soy products, etc.

3. Fr Dried beans and products: mung beans, soybeans, red beans, soy milk, oily tofu, tofu, other soy products, etc.esh milk: unprocessed milk, goat milk, horse milk, etc.

4. Dairy products: yogurt, cheese, milk powder, cream, butter butter, ice cream, etc.

5. Edible fungi: shiitake mushrooms, fresh mushrooms, oyster mushrooms, straw mushrooms, fungus, kelp, seaweed, etc.

6. Yellow-green vegetables: Chinese cabbage, spinach, cabbage, green cabbage, water spinach, rapeseed, mustard greens, celery, taikecai, chicken hair greens, lettuce, kohlrabi, etc.

7. Root vegetables: red heart potato, yellow heart potato, potato, fresh bamboo shoot, radish, carrot, lotus root, taro, ginger, etc.

8. Melon vegetables: winter melon, cucumber, pumpkin, loofah, gourd, etc.

9. Onion and garlic vegetables: garlic, onion, onion, leeks, garlic sprouts, etc.

10. Nightshade vegetables: eggplants, tomatoes, sweet green peppers, peppers, etc.

11. Fresh fruits: apple, pear, tangerine/orange, banana, grape, peach, watermelon, melon, pomegranate, hawthorn, jujube, pineapple, strawberry, sugar cane, persimmon, kiwi, apricot, plum, mango, etc.

12. Fried foods: fried chicken, fried noodles tow fish, fried noodles with pork chops, spring rolls, fried dough sticks, etc.

13. Preserved food: cured meat, pickled fish, pickles, mustard, fermented bean curd, salted duck eggs, songhua eggs, etc.

14. Sweets: cakes/pastry, sweet bread, biscuits, red bean soup, mung bean soup, etc.

15. Nuts: walnuts, melon seeds, peanuts, almonds, figs, almonds, dried dates, pistachios, hazelnuts, cashews and other hard fruits

16. Poultry eggs: eggs, duck eggs, goose eggs, quail eggs, etc.

| **A** General situation | |
| --- | --- |
| A1 Gender: 1 male 2 female |  |
| A2 Date of birth: (Fill in four digits, such as 1983,8) |  |
| A3 weight: kg |  |
| A4 height： cm |  |
| A5 Nationality：1 Han 2 Uighur 3 Hui 4 Kazak 5 Mongolian 6 Xibe 7 Kirgiz 8 Tatar 9 others |  |
| A6 Local residence years (limited to more than one year): |  |
| A7 birthplace: |  |
| A8 Marital status: 1 unmarried 2 married 3 divorced 4 widowed 5 other |  |
| A9 Education level: 1 no school 2 elementary school 3 junior high school 4 high school/technical school/technical school 5 junior college 6 university and above |  |
| A10 occupation:1 Cadres and business leaders 2 Professional and technical personnel 3 General staff 4 Agriculture and forestry workers 5 Animal husbandry workers 6 Individual industrial and commercial households 7 Retired or housework 8 Others |  |
| A11 Sleep time: 15 hours and below 2 6-7 hours 3 7-8 hours 4 8 hours or more |  |
| A12 Social Medical Insurance:1 None 2 Urban basic medical insurance 3 Public medical care 4 Labor insurance medical care 5 Cooperative medical care 6 Others |  |
| A13 How much is your personal annual income (net income in rural areas, cash income in cities): 1 Less than 7,000 yuan 2 7,000 yuan-20,000 yuan 3 More than 20,000 yuan |  |
| **B Smoking and drinking** | |
| B1 Do you smoke: 1 No (skip to B6) 2 Smoking (1 cigarette a day for up to half a year) 3 Quit smoking |  |
| B2 If you have ever smoked, the age at which you started smoking: |  |
| B3 If you quit smoking, quit smoking age： |  |
| B4 How long do you smoke： |  |
| **B5** How many cigarettes (grams) you smoked on average per day before smoking or quitting: (Calculate the number of grams per day for pipes and hand-rolled cigarettes) |  |
| B6 Do you drink alcohol: 1 No (skip to B12) 2 Yes 3 quit drinking |  |
| B7 How old did you start drinking: |  |
| B8 How many years have you been drinking: |  |
| B9 The type of wine you usually drink is the most: 1 white wine 2 wine 3 beer 4 rice wine 5 others |  |
| B10 You drink alcohol a few times a week on average:1 Once every 2-3 weeks 2 1-2 times a week 3 3-4 times a week 4 5 times a week and above |  |
| **B11** What is the average amount of alcohol you drink each time (grams): |  |
| B12 Do you often drink horse milk wine: 1 No (skip to C1) 2 Occasionally 3 Often |  |
| B13 How many bowls of martinet do you usually drink each month: |  |
| **C** Tea (at least once a week for more than 3 months) | |
| C1 Do you drink tea: 1 No (skip to C4) 2 Yes |  |
| C2 How many times do you drink tea a week: 1 1-2 times a week 2 3-4 times a week 3 5 times a week and above |  |
| C3 What kind of tea do you usually like to drink: 1 light 2 moderate 3 strong tea |  |
| C4 Do you drink milk tea: 1 No (skip to D1) 2 Yes |  |
| C5 How old did you start to develop the habit of drinking milk tea: |  |
| How many bowls of milk tea do you drink every day: 1 1 bowl every 2-3 days 2 1-2 bowls a day 3 3-4 bowls a day 4 5 bowls and above every da |  |
| C7 What kind of milk tea do you usually like to drink: 1 light 2 moderate 3 strong tea |  |
| **D** diet | |
| D1 Do you usually like to eat breakfast: 1 No 2 Occasionally 3 Often |  |
| D2 Do you usually like to overeating: 1 No 2 Occasionally 3 Often |  |
| D3 Do you usually like to eat and drink before going to bed: 1 No 2 Occasionally 3 Often |  |
| D4 Do you usually like spicy food: 1 No 2 Occasionally 3 Often |  |
| D5 Which type of meat do you usually eat the most: 1 Beef 2 Lamb 3 Horse meat 4 Chicken 5 Other meats |  |
| D6 Do you eat barbecued meats (intestines, meat): 1 No (skip to D9) 2 Yes |  |
| D7 How many catties do you usually eat per month: 1 less than 1 kg per month 2 1-2 kg per month 3 2 kg and above per month |  |
| D8 Barbecue: 1 Eat every day 2 4-6 times/week 3 1-3 times/week 4 Eat several times a month 5 Rarely eat |  |
| D9 Do you eat fresh meat: 1 No (skip to D12) 2 Yes |  |
| D10 How many catties of fresh meat do you usually eat per month: 1 Less than 1 kg per month 2 1-2 kg per month 3 Over 2 kg per month |  |
| D11 Fresh meat: 1 Eat every day 2 4-6 times/week 3 1-3 times/week 4 Eat several times a month 5 Rarely eat |  |
| D12 Do you eat salted or air-dried meat: 1 No (skip to D15) 2 Yes |  |
| D13 How many catties of salted or dried meat do you usually eat per month: 1 Less than 1 kg per month 2 1-2 kg per month |  |
| D14 Salted or dried meat: 1 Eat every day 2 4-6 days/week 3 1-3 days/week 4 Eat several times a month 5 Rarely eat |  |
| D15 Yellow-green vegetables: 1 Eat every day 2 4-6 times/week 3 1-3 times/week 4 Eat several times a month 5 Rarely 6 Do not eat |  |
| D16 Onions and garlic: 1 Eat 2 4-6 times/week 3 1-3 times/week 4 Eat several times a month 5 Rarely eat 6 Do not eat |  |
| D17 Roots: 1 Eat 2 4-6 times/week 3 1-3 times/week 4 Eat several times a month 5 Rarely eat 6 Do not eat |  |
| D18 Edible fungi: 1 Eat 2 4-6 times/week 3 1-3 times/week 4 Eat several times a month 5 Rarely eat 6 Do not eat |  |
| D19 Fried type: 1 Eat 2 4-6 times/week 3 1-3 times/week 4 Eat several times a month 5 Rarely eat 6 Don’t eat |  |
| D20 Pickled vegetables: 1 Eat every day 2 4-6 times/week 3 1-3 times/week 4 Eat several times a month 5 Rarely eat 6 Don’t eat |  |
| D21 Sweets: 1 Eat every day 2 4-6 times/week 3 1-3 times/week 4 Eat several times a month 5 Eat rarely 6 Don’t eat |  |
| D22 Fresh fruits: 1 Eat every day 2 4-6 times/week 3 1-3 times/week 4 Eat several times a month 5 Rarely eat 6 Do not eat |  |
| D23 Nuts: 1 Eat every day 2 4-6 days/week 3 1-3 days/week 4 Eat several times a month 5 Eat rarely 6 Don’t eat |  |
| D24 Fresh milk: 1 Eat every day 2 4-6 times/week 3 1-3 times/week 4 Eat several times a month 5 Eat rarely 6 Don’t eat |  |
| D25 Dairy products: 1 eat 2 4-6 times a week 3 1-3 times a week 4 eat several times a month 5 rarely eat 6 do not eat |  |
| D26 Poultry and eggs: 1 Eat every day 2 4-6 times/week 3 1-3 times/week 4 Eat several times a month 5 Rarely eat 6 Do not eat |  |
| D27 Drinking water situation: 1 tap water 2 shallow well water (hand pressure well) 3 deep well water 4 rainwater and river water 5 mountain spring water 6 others |  |
| **E** Medical history and family history (diagnosed by hospitals above the township health center) | |
| E1 Do you have high blood pressure: 1 No 2 Yes |  |
| E2 Do you have hyperlipidemia: 1 No 2 Yes |  |
| E3 Have you ever been diagnosed with gallbladder disease: 1 No 2 Gallbladder stones 3 Gallbladder polyps 4 Acute or chronic cholecystitis 5 Gallbladder cancer |  |
| E4 Do you have diabetes: 1 No 2 Yes |  |
| E5 Do your parents have gallbladder disease: 1 No 2 Gallbladder stones 3 Gallbladder polyps 4 Acute or chronic cholecystitis 5 Gallbladder cancer |  |
| E6 Do your brothers and sisters suffer from gallbladder disease:  1 No 2 Gallbladder stones 3 Gallbladder polyps 4 Acute or chronic cholecystitis 5 Gallbladder cancer |  |
| E7 Do your grandparents and grandparents suffer from gallbladder disease:  1 No 2 Gallbladder stones 3 Gallbladder polyps 4 Acute or chronic cholecystitis 5 Gallbladder cancer |  |
| E8 Does your child have gallbladder disease: 1 No 2 Gallstones 3 Gallbladder polyps 4 Acute or chronic cholecystitis 5 Gallbladder cancer |  |
| **F** Female factor (If you are unmarried, skip to F8) | |
| F1 Do you have children: 1 No (skip to F5) 2 Yes |  |
| F2 How many children have you had: |  |
| F3 The age of your first child: |  |
| F4 The feeding method of the child after birth: 1 breastfeeding 2 artificial feeding 3 mixed feeding |  |
| F5 How many times have you been pregnant (including miscarriage): |  |
| F6 If menopause, your menopausal age (Premenopausal, skip to F7): |  |
| F7 Are you oral contraceptives: 1 No 2 Occasionally 3 Often |  |
| F8 Do you suffer from irregular menstruation: 1 No 2 Yes |  |
| F9 Your age at menarche (first menstrual period): |  |
| Ultrasound examination results (Completed by ultrasound doctor): |  |
